# Hydrochemistry of the Hot Springs in Western Sichuan Province Related to the Wenchuan *M*_*S*_ 8.0 Earthquake

**DOI:** 10.1155/2014/901432

**Published:** 2014-05-05

**Authors:** Zhi Chen, Jianguo Du, Xiaocheng Zhou, Li Yi, Lei Liu, Chao Xie, Yueju Cui, Ying Li

**Affiliations:** ^1^School of Earth and Space Sciences, University of Science and Technology of China, Hefei 230026, China; ^2^CEA Key Laboratory of Earthquake Prediction (Institute of Earthquake Science), China Earthquake Administration, Beijing 100036, China

## Abstract

Hydrogeochemistry of 32 hot springs in the western Sichuan Province after the Wenchuan *M*_*S*_ 8.0 earthquake was investigated by analyzing the concentrations of cation and anion and the isotopic compositions of hydrogen and oxygen. The water samples of the hot springs were collected four times from June 2008 to April 2010. Hydrogeochemical data indicated the water samples can be classified into 9 chemical types. Values of **δ**D and **δ** ^18^O indicated that the spring waters were mainly derived from meteoric precipitation and affected by water-rock interaction and mixture of deep fluids. Concentrations of K^+^and SO_4_^−^ of the samples from the Kangding district exhibited evident increases before the Wenchuan earthquake, indicating more supplement of deep fluids under the increase of tectonic stress. The chemical and isotopic variations of the water samples from the area closer to the epicenter area can be attributed to variation of regional stress field when the aftershock activities became weak.

## 1. Introduction

Great earthquakes usually associate with the physical-chemical variations of groundwater and hot springs. The short-term hydrogeochemical precursors for earthquake [1–4], co-seismic response of hydrochemistry [5, 6] and postseismic geochemical and isotopic changes of hot springs have been reported [7–12].

The observed geochemical anomalies related to earthquakes are usually attributed to the alteration of groundwater in the circulating system under the action of increasing crustal stress before and after earthquakes [6, 7, 13, 14]. For understanding the hydrogeochemical anomalies related to earthquakes, some genetic mechanism models have been proposed, such as increased solubility of rocks under increased pressure and release of ions from rocks into water [15], pore collapse with fluids expulsion [8], the water-rock interactions at the enhanced reactive surfaces [16], aquifers rupture with mixing of different fluids [6] and expulsion of deep fluids by tectonic pumping [1].

Hydrogeochemical survey of hot springs in western Sichuan before the *M*_*S*_ 8.0 Wenchuan earthquake mainly focused on the origin of waters, the pollutions, the chemical classifications and the heat reservoirs in the sites surrounding Kangding county [17–25].

The *M*_*S*_ 8.0 earthquake occurred in Wenchuan county, Sichuan province, Southwest China on 12 May, 2008, following by hundreds of aftershocks with magnitudes higher than 3.0.

In order to investigate the hydrogeochemical characteristics of the springs related to the seismic activities, the hydrogeochemical survey of 32 hot springs in the western Sichuan were performed four times from June 2008 to April 2010.

## 2. Geological Setting

**Figure 1:**
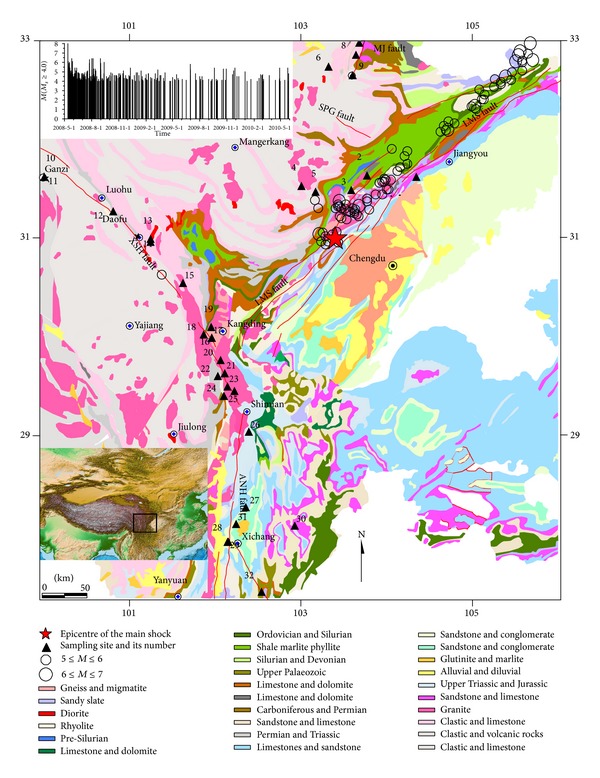
Schematic map of geology in western Sichuan Province, showing the sample locations, and earthquake epicenters, the insert figure is time-magnitude plot for aftershocks with *M*_*S*_ ≥ 4, red lines are active faults.

The investigated area is located at the eastern margin of the Tibetan Plateau, in which there are four major fault zones named as Minjiang fault (MJF), Longmenshan fault (LMSF), Xianshuihe fault (XSHF) and Anninghe fault (ANHF) zones (Figure 1) where earthquakes frequently occurred [26].Triassic littoral-neritic clastic rocks interbeded with carbonate and intrusive granite are exposed in the west of the LMSF and ANHF zones, but the strata from Late Paleozoic to Cenozoic are widespread exposed in the LMSF and ANHF zones and on the east of the fault zones, and granite is found in the intersection of the LMSF, XSHF and ANHF zones [27–30]. The fault zones act as the important passage for upward migration of thermal fluids from the deep earth, which is indicated by distribution of many hot springs in the MJF, LMSF, XSHF and ANHF zones (Figure 1). Historically, a number of great earthquakes (*M*_*S*_ > 7.0) have occurred in the investigated area since 1800. For instance, the 1850 *M*_*S*_ 7.5 Xichang earthquake occurred in the ANHF zone, two *M*_*S*_ 7.2 earthquakes of 16 and 23 August 1976 in the MJF zone, the *M*_*S*_ 7.5 one of 25 August 1933 in the LMSF zone, and the *M*_*S*_ 7.5 one of 1955 and the *M*_*S*_ 7.9 one of 1973 in the XSHF zone [12]. The *M*_*S*_ 8.0 Wenchuan earthquake resulted in a 285 km surface rupture zone along the pre-existing Yingxiu-Beichuan, Guanxian-Anxian and Qingchuan faults, with the maximum vertical surface offset of about 6.2 m [31].

## 3. Methods

Water samples were repeatedly collected four times (in June and October 2008, June 2009, and April 2010) at 32 sites of spas, wells, and springs (Figure 1) in the MJF, LMSF, XSHF, and ANHF zones. The samples were sealed and stored in 500 mL glass bottles. The values of temperature were measured with a digital thermometer with an error of ±1% in the field. Isotopic compositions of *δ*D and *δ* ^18^O were measured with a Picarro L1102 mass spectrometer in the Laboratory of Gas Geochemistry, Institute of Geology and Geophysics, Chinese Academy of Sciences in Lanzhou, and the errors are 0.5‰ for *δ*D and 0.1‰ for *δ* ^18^O, respectively. The concentrations of cations (K^+^, Na^+^, Mg^2+^, and Ca^2+^) and anions (F^−^, Cl^−^, Br^−^, NO_3_^−^, and SO_4_^2−^) were determined with the Dionex ICS-900 ion chromatography (reproducibility within ±2%) that is installed in the Institute of Earthquake Science, China Earthquake Administration. The CO_3_^2−^ and HCO_3_^−^ concentrations were measured by the standard titration procedures with a ZDJ-100 potentiometric titrator (reproducibility within ±2%). The ion balance (ib) was calculated according to the following equation [11]:

(1) $$\begin{array}{c} \text{ib}\left(\%\right)=\frac{\sum \text{cations}-\sum \text{anions}}{\left(\sum \text{cations}+\sum \text{anions}\right)\times 0.5}\times 100. \end{array}$$

## 4. Results

The physicochemical parameters of the 32 springs were listed in Table 1. No water samples were collected from the springs of nos. 2 and 24–26 in June 2008 because the springs were damaged by the Wenchuan earthquake. No samples were collected from the springs nos. 8 and 29 in April 2010 and the spring no. 28 in June 2009 and April 2010 because of the postearthquake reconstruction. The ion balance values of measured chemical data were calculated to be less than 5% (Table 1).

### 4.1. Physicochemical Parameters of the Springs in the LMSF Zone

The water samples from in the LMSF zone have *δ* ^18^O and *δ*D values between −16.34‰ and −6.18‰, −116.65‰ and −61.66‰, respectively. The temperatures and TDS ranged from 10.5 to 58.0°C and 151.69 to 1569.81 mg/L. The concentrations of Na^+^, Ca^2+^ and Mg^2+^ varied from 29.82 to 202.49 mg/L, 4.74 to 252.78 mg/L, and 0.80 to 128.73 mg/L, respectively. The concentrations of Cl^−^, SO_4_^2−^, and HCO_3_^−^ varied from 2.31 to 64.74 mg/L, 21.33 to 878.08 mg/L, and 14.63 to 310.70 mg/L respectively (Table 1).

### 4.2. Physicochemical Parameters of the Springs in the MJF Zone

Fifteen water samples were collected from the sites nos. 6–9 in the MJF zone, of which the *δ* ^18^O and *δ*D were between −15.34% and −13.20%, −112.23% and −102.20%, the values of temperatures and TDS ranged from 8.8 to 21.7°C and 505.34 to 1657.61 mg/L. The concentrations of Na^+^, Ca^2+^, and Mg^2+^ ranged from 8.75 to 25.23 mg/L, 49.54 to 183.57 mg/L, and 23.23 to 183.57 mg/L, and the concentrations of Cl^−^, SO_4_^2−^, and HCO_3_^−^ from 0.93 to 10.78 mg/L, 3.56 to 17.78 mg/L, and 313.61 to 1324.27 mg/L separately (Table 1).

### 4.3. Physicochemical Parameters of the Springs in the XSHF Zone

Sixty-two water samples were collected from the sites nos. 10–25 in the XSHF zone, of which *δ* ^18^O and *δ*D varied from −18.84‰ to −10.04‰ and −141.98‰ to −78.77‰. The values of temperatures and TDS ranged from 27.8 to 83.0°C and 427.53 to 2159.43 mg/L, respectively. The concentrations of Na^+^, Ca^2+^, and Mg^2+^ ranged from 35.14 to 679.23 mg/L, 4.38 to 50.10 mg/L, and 0.12 to 45.71 mg/L, respectively. The concentrations of Cl^−^, SO_4_^2−^, and HCO_3_^−^ ranged from 2.35 to 336.16 mg/L, 6.56 to 161.87 mg/L, and 62.48 to 1894.64 mg/L, respectively (Table 1).

### 4.4. Physicochemical Parameters of the Springs in the ANHF Zone

Twenty-four water samples were collected from the sites nos. 26–32 in the ANHF zone, of which the *δ* ^18^O and *δ*D were between −11.64‰ and −96.10‰, −110.47‰ and −102.20‰. The temperatures of the spring waters were between 21.1 and 56.9°C, the TDS were between 206.70 and 1212.86 mg/L. The concentrations of Na^+^, Ca^2+^, and Mg^2+^ ranged from 50.17 to 213.50 mg/L, 2.00 to 81.48 mg/L, and 0.00 to 29.43 mg/L, and the concentrations of Cl^−^, SO_4_^2−^, and HCO_3_^−^ from 1.91 to 136.74 mg/L, 10.94 to 257.26 mg/L, and 81.95 to 449.06 mg/L, respectively (Table 1).

## 5. Discussion

### 5.1. Chemical Types of the Waters and Their Origins

#### 5.1.1. The Chemical Types

**Figure 2:**
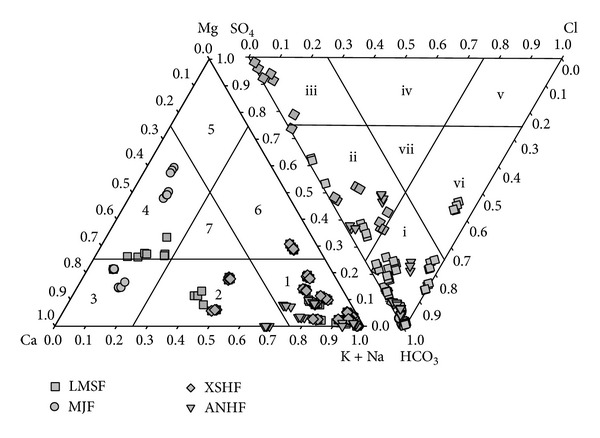
Ternary plots of the cation and anion for the waters.

The triangular diagrams in Figure 2 illustrated the proportions of the anionic (Cl^−^, SO_4_^2−^, and HCO_3_^−^) and cationic (K^+^ + Na^+^, Mg^2+^, and Ca^2+^) concentrations of the water samples from the 32 sites. The anions and cations of the water samples were mainly distributed in blocks i, ii, and iii, indicating that Na^+^, Ca^2+^, SO_4_^2−^, and HCO_3_^−^ were the main chemical composition for most of the samples. Based on the Shoka Lev's classification method, the water samples were classified into 9 chemical types according to the main chemical composition, which are Na(Ca)-HCO_3_(SO_4_), Na(Mg)-HCO_3_(SO_4_), Ca(Na)-HCO_3_(SO_4_), Mg(Ca)-SO_4_, Ca(Mg)-SO_4_, Ca(Mg)-HCO_3_, Mg(Ca)-HCO_3_, Na-Cl(HCO_3_), and Ca(Na)-SO_4_(HCO_3_) (Table 1). The cations of the samples of the springs nos. 1–5 occur in the LMSF zone were mainly distributed in the blocks 1, 2, and 4, and the anions were distributed in the blocks i and ii, which together formed 4 chemical types of Na(Ca)-HCO_3_(SO_4_), Ca(Na)-HCO_3_(SO_4_), Mg(Ca)-SO_4_, and Ca(Mg)-SO_4_. The cations of the samples of the springs nos. 6–9 occur in the MJF zone were mainly distributed in the blocks 3 and 4, the anions were distributed in the block i, and the chemical types for the samples were Ca(Mg)-HCO_3_ and Mg(Ca)-HCO_3_. Both of the cations and anions of the samples of the springs nos. 26–32 occur in the ANHF zone were distributed in the blocks i-ii and 1-2, these samples have the similar chemical type of Na(Ca)-HCO_3_(SO_4_). Half of the samples including the samples of the springs nos. 10–25 occur in the XSHF zone, the cations of these samples were distributed in the blocks 1, 2, and 5, with anions in the blocks i, ii, iii, and vi, and the chemical types were Na(Ca)-HCO_3_(SO_4_), Na(Mg)-HCO_3_(SO_4_), Na-Cl(HCO_3_), and Ca(Na)-SO_4_(HCO_3_).

#### 5.1.2. The Origins

**Figure 3:**
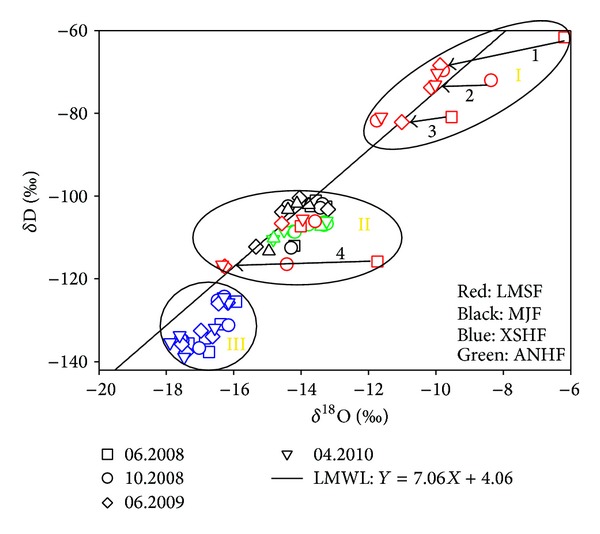
Diagram of *δ* ^18^O versus *δ*D of the waters. LMWL stands for the Local Meteoric Water Line: *δ*D = 7.06 *δ* ^18^O − 4.06.

*(1) *δ*D and *δ** ^*18*^ *O.* The values of *δ* ^18^O and *δ*D of the 32 springs were plotted along the local meteoric water line (LMWL: *δ*D = 7.06 *δ* ^18^O + 4.06) with different extent shifts from the LMWL (Figure 3), which indicated that the spring waters were mainly originated from meteoric water and with different extent alteration. The values of *δ* ^18^O and *δ*D were plotted in three regions, the region I includes the samples of the springs no.1–3, with values of *δ*D and *δ* ^18^O ranged from −82.13‰ to −61.66‰ and −11.75‰ to −6.18‰, the region III includes all the samples from the XSHF zone (springs nos. 10–25), with values of *δ*D and *δ* ^18^O between −141.98‰ and −78.77‰, −18.84‰ and −10.04‰, respectively, and the others in the region II, with the values of *δ*D and *δ* ^18^O ranging from −116.81‰ to −96.10‰ and −16.34‰ to −11.64‰ (Table 1). The values of *δ*D and *δ* ^18^O of the springs nos.10–25 from the higher mountain area were more negative, while those of the springs nos. 1–3 collected in the lower altitude region were less negative, which is constant with the previous results [32].

*(2) Na(Ca)-HCO*_*3*_ *(SO*_*4*_ *), Na(Mg)-HCO*_*3*_ *(SO*_*4*_ *), and Na-Cl(HCO*_*3*_ *) Waters.* The water samples of the springs nos. 4-5, 10–23 and 25–32 were belong to these types (Table 1). The springs nos. 4-5, 13–23 and 25 occur in granite, while the springs nos. 10-11 in Late Triassic clastic rock interbeded with volcanic rocks. The spring no. 12 is found in Late Permian volcanic tuff, while the spring no. 26 in Sinian pyroclastic rock. The spring nos. 27–29 and 31 are found in Cretaceous clastic rock, the spring no. 30 in Early Triassic sandstone, and the spring no. 32 in Paleozoic mixed layer (Figure 1). The springs nos. 4-5, 10–23 and 25–32 occur in the rocks enriched in feldspar resulting in similar higher concentrations of Na^+^ and HCO_3_^−^ because of rock-water interaction. In addition, the samples from the springs nos. 5, 19, 23, and 32 were characterized by the similar concentrations of Ca^2+^, Mg^2+^, and SO_4_^2−^ (Table 1), which should be attributed to the water-rock interactions between groundwater and Devonian carbonate (Figure 1). Cl^−^ is known to be conservative and derive from the deep earth mainly [33, 34]. The chemical type for the samples from the spring no. 16 was Na-Cl (HCO_3_), with the Cl^−^ concentration as 328.54 mg/l (Table 1), which suggested the input of deep fluid. Meanwhile, the high ^3^He/^4^He ratio (between 1.43 and 3.73RA, RA = 1.39 × 10^−6^) [30] together with the high temperatures (between 80.0°C and 70.2°C) also are the evidences for the upwelling of the deep-earth fluids into the spring (Table 1). 

*(3) Ca(Na)-HCO*_*3*_ *(SO*_*4*_ *), Ca(Mg)-HCO*_*3*_ *, Mg(Ca)-HCO*_*3*_ *, and Ca(Na)-SO*_*4*_ *(HCO*_*3*_ *) Waters*. These included the samples from the springs nos.1, 6–9, and 24 (Table 1). The spring no. 1 occurs in Jurassic carbonate interbedded with sandstone. The springs nos. 6 and 9 occur in Late Triassic clastic rock interbedded with carbonate. The springs nos. 7 and 8 occur in Middle Triassic clastic rock interbedded with carbonate, and the spring no. 24 is found in Carboniferous carbonate (Figure 1). The springs nos. 1, 6–9, and 24 occur in strata enriched in carbonate, so that the main components of the waters were Ca^2+^, Mg^2+^ and HCO_3_^−^ because of the interaction between the groundwater and carbonate. The samples of the springs nos. 8 and 9 have the same chemical type of Mg (Ca)-HCO_3_, and similar Ca/Mg ratios (0.54 and 0.86, Table 1), which can be attributed to the dissolution of calcite combined with the low temperatures of the springs nos. 8 and 9 (8.8°C and 9.0°C respectively) (Table 1).

*(4) Mg(Ca)-SO*_*4*_ *and Ca(Mg)-SO*_*4*_ *Waters.* The springs nos. 2 and 3 occur in Devonian to Silurian pyrite-bearing sale and marlite (Figure 1), from which the samples had higher SO_4_^2−^ concentration (878.08 mg/L and 812.50 mg/L, resp.) and TDS (1571.81 mg/L and 1195.08 mg/L, resp.), with the chemical types of Mg (Ca)-SO_4_ and Ca (Mg)-SO_4_ (Table 1), indicating interaction between carbonate and groundwater enhanced by the sulfate from the oxidation of pyrite [35].

### 5.2. Chemical Variations of the Spring Waters Associated with Seismic Activities

#### 5.2.1. Temporal Variations

The hydrochemical data of the water samples showed evident trend of temporal variations.

The geochemical parameters of the samples nos. 1, 3–5 from the LMSF zone and the samples nos. 15 and 20 from the XSHF zone varriedby more than 20% one month after the Wenchuan earthquake, and similar variations happened after October 2008. The concentrations of Na^+^, Ca^2+^, Cl^−^, SO_4_^2−^, and TDS of the sample of the spring no. 1 decreased by 42%, 39%, 53%, 31%, and 35%, respectively; for the sample number 2, the decreasing amplitudes for the concentrations of Na^+^, Mg^2+^, Ca^2+^, Cl^−^, SO_4_^2−^, and TDS were 23%, 45%, 25%, 38%, 22%, and 33%, separately. The concentrations of Mg^2+^, Ca^2+^, HCO_3_^−^, and TDS of the sample from the spring no. 3 decreased by 33%, 42%, 95%, and 35%, respectively. The concentrations of Na^+^, HCO_3_^−^, and TDS of the sample of the spring no. 4 decreased by 67%, 33% and 57%, respectively. For the sample of the spring no. 5, the concentrations of Na^+^, HCO_3_^−^ and TDS decreased by 25%, 41%, and 26%, respectively. The concentrations of Na^+^ and SO_4_^2−^ of sample of the spring no. 20 decreased by 28% and 26%, respectively. For the sample of the spring no. 15, however the concentrations of Na^+^, HCO_3_^−^ and TDS increased in 17 April 2010, with the amplitudes of 53%, 38%, and 38%, respectively. Meanwhile, the *δ* ^18^O-*δ*D plots of the samples from the springs nos.1, 3, and 4 approached the LMWL (Local Meteoric Line) after sizeable shift in June 2008, and the similar variation happened for the spring no. 2 after October 2008 (Figure 3), which indicated gradual input decrease of deep-earth fluids that formed by water-rock reaction after the Wenchuan earthquake

**Figure 4:**
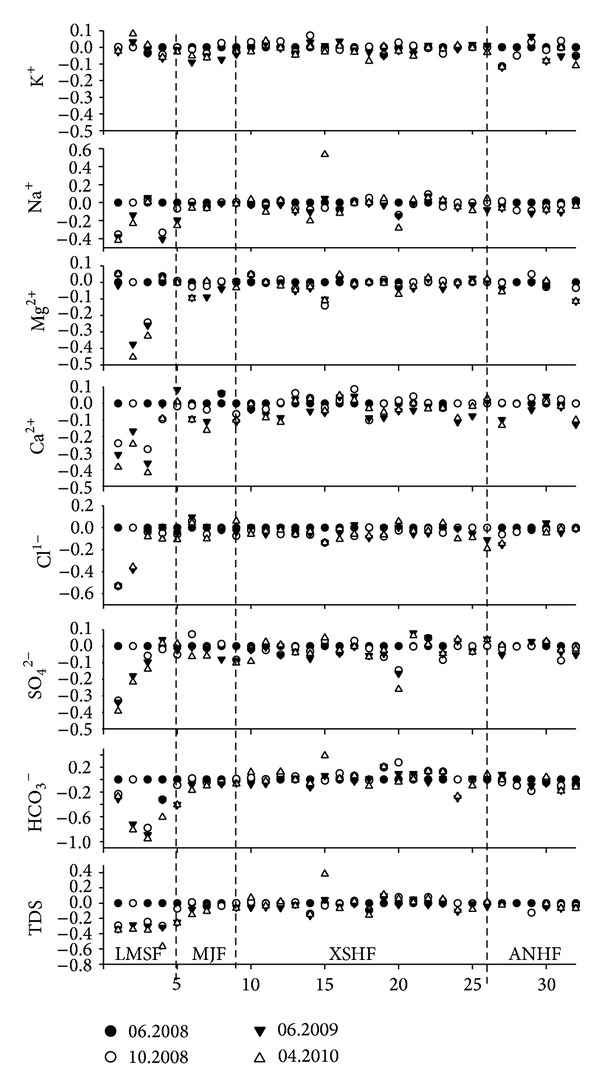
Temporal variations of the geochemical parameters of the 32 hot spring waters after the Wenchuan earthquake. The abscissas indicate the numbers of the samples and the ordinates indicate the ratios of each batch of samples with the first batch of samples.

The supplement of deep water characterized by higher mineralization and enriched in *δ* ^18^O is considered as a main factor for controlling pre- and co-seismic geochemical variations of the groundwater and springs [2, 6, 7, 11, 13, 14, 16, 36, 37]. The Coulomb stress in the middle-north segment of the LMSF zone, the southeast segment of the XSHF zone, and the south segment of the MJF zone had been enhanced, which resulted in the Wenchuan *M*_*S*_ 8.0 earthquake [38, 39], and the geochemical variations of the samples from the springs nos. 1–5 and 20. Meanwhile, the input decrease of deep-earth water during the sampling period in which seismic activity decreased gradually. 747 aftershocks with *M*_*S*_ higher than 4.0 occurred in the three fault zones, and most of which (including all the 12 events with amplitude ranged from 6.0 to 6.4) occurred before September 10, 2008 (China Earthquake Network Center, Figure 1). As the aftershock activities became weak, the TDS values of the samples from the springs nos. 1–5 and 20 decreased gradually to the normal value (Figure 4), indicating the supplement decrease of the deep-earth fluid as the Coulomb stress being released after the events as indicated by the data of oxygen and hydrogen isotope compositions (Figure 3).

Notably, the concentrations of Na^+^, HCO_3_^−^, and TDS of the sample of the spring no. 15 increased by 53%, 38%, and 38% on April 17, 2010 (Figure 4), which was closely related to the 28 April 2010 an *M*_*S*_ 5.4 aftershock 15 kilometers epicenter distance to the spring no. 15 (Figure 1). Therefore, it can be infered that the hydrogeochemical variations of the spring no. 15 might be the short-term hydrogeochemical precursors for the *M*_*S*_ 5.4 event.

**Figure 5:**
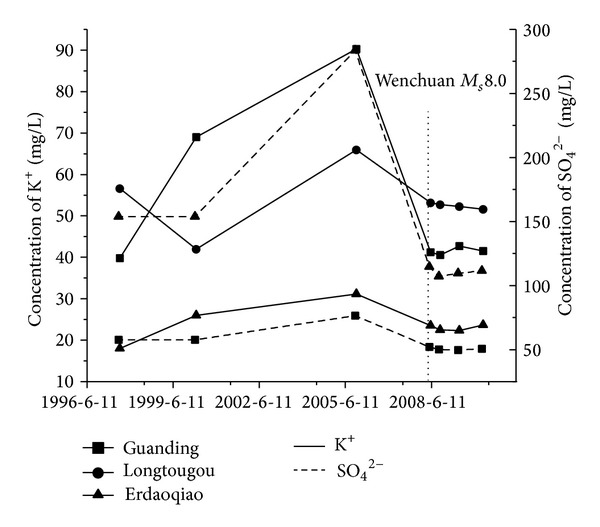
Temporal variations of concentration of K^+^ and SO_4_^2−^. Black squares: waters collected from the Guanding spring; black cycles: waters collected from the Longtougou spring; black triangles: waters collected from the Erdaoqiao spring; the dashed vertical line: the time of the occurrence of the Wenchuan earthquake.

The geochemical parameters of the samples from the springs nos. 16-17 and 19 about 200 kilometers showed evident increase trend before the Wenchuan earthquake [20, 24, 25]. The K^+^ concentrations of the samples of the springs nos. 16-17 and 19 increased by 19.3% to 54.4% before the main shock, and rapidly decreased to the normal values after the main shock. The concentrations of SO_4_^2−^ for the samples from the springs nos. 16 and 19 increased by 32.0% and 59.6% respectively before the earthquake, and then dropped to the normal value after the main shock (Figure 5). The springs nos. 16-17 and 19 occur in the intersection of the LMSF, XSHF and ANHF zones where the extrusion stress increased yearly from 2004 to 2007 before the Wenchuan earthquake [40, 41]. Therefore, the increase of K^+^ and SO_4_^2−^ concentrations for the samples of the springs nos.16-17 and 19 could be attributed to the more supplement of the deep fluids with higher concentrations of K^+^ and SO_4_^2−^ [42], The ^3^He/^4^He ratios of the samples from the springs nos. 16-17 and 19 increased before the Wenchuan earthquake, but showed a remarkable drop after the shock, and then became normal indicating the more supplement of mantle-derived fluids before the main shock [30].

#### 5.2.2. Spatial Variations

The geochemical and isotopic compositions of the samples from the springs nos. 1–5 occur in the LMSF zone performed evident variation after the Wenchuan earthquake, with the highest amplitude as 95% for HCO_3_^−^ of the sample from the spring no. 5. However, for the other samples from the other fault zones, just the samples from the springs nos. 15 and 20 presented obvious geochemical variations, with the highest amplitude as 53% for Na^+^ of the sample from the spring no. 15 (Figure 4). The Wenchuan earthquake happened in the LMSF zone, which enhanced the Coulomb stress of the zone [38, 39], and with most of the aftershocks occurred there (Figure 1). For the springs nos. 1–5 occurred in the LMSF zone, the epicenter distances are between 50 and 110 kilometers, which are smaller than those of the hot springs nos. 6-32 (between 190 and 410 km). Therefore, the variation of the tectonic stress induced by the Wenchuan earthquake and the aftershocks may resulted in, the geochemical and isotopic variations for the springs nos. 1–5 after the Wenchuan earthquake.

## 6. Conclusions

120 water samples of the 32 hot springs in the western Sichuan have been analyzed after the Wenchuan earthquake. The following conclusions can be drawn.

1. The waters of the 32 springs were mainly derived from meteoric precipitation. Because of the isotopic elevation effect, the *δ*D and *δ* ^18^O of the samples from the higher mountain area were lower, but those of samples from the lower altitude region were higher. all of the analyzed water samples from the 32 springs can be classified into nine chemical types: Na(Ca)-HCO_3_(SO_4_), Na(Mg)-HCO_3_(SO_4_), Ca(Na)-HCO_3_(SO_4_), Mg(Ca)-SO_4_, Ca(Mg)-SO_4_, Ca(Mg)-HCO_3_, Mg(Ca)-HCO_3_, Na-Cl(HCO_3_), and Ca(Na)-SO_4_(HCO_3_), which were mainly controlled by water-rock interaction and the input of deep fluid.
2. The concentrations of K^+^ and SO_4_^2−^ of the samples of the springs nos.16-17 and 19 in the intersection of LMSF, XSHF, and ANHF varied evidently, with the amplitude ranging from 19.3% to 59.6% before and after the Wenchuan earthquake which may be attributed to the interfusion of the deep fluids with high K^+^ and SO_4_^2−^ induced by the increased tectonic stress.
3. The hydrogeochemical variations of the springs closer to the epicenter performed more obviously after the Wenchuan earthquake. As the aftershock activities became weak, the geochemical parameters of the samples from the springs nos. 1–5, 15, and 20 located in the regions where the tectonic stress was enhanced before the Wenchuan *M*_*S*_ 8.0 earthquake decreased by more than 20%, and with the *δ* ^18^O the LMWL, which may be related to the change of tectonic stress when the aftershock activities became weak.

## Figures and Tables

**Table 1:** Physicochemical parameters of the 32 hot spring waters.

Number	Site	*L* (E)	*B* (N)	Date	*T* °C	TDS mg/L	K^+^ mg/L	Na^+^ mg/L	Ca^2+^ mg/L	Mg^2+^ mg/L	Cl^−^ mg/L	SO_4_^2−^ mg/L	CO_3_^2−^ mg/L	HCO_3_^−^ mg/L	ib %	*δ* ^ 18^O ‰	*δ*D ‰	Chemical type
The LMSF zone																		
1	Sangzao Spring	104.35	31.61	11.06.2008	19.0	557.43	2.67	64.78	73.27	12.27	64.74	108.24	0.00	229.38	−0.01	−6.18	−61.66	CaNa–HCO_3_SO_4_
				01.11.2008	19.1	394.07	2.68	41.92	55.57	12.90	30.39	72.65	0.00	175.98	0.02	−9.79	−69.63	CaNa–HCO_3_SO_4_
				01.07.2009	19.7	367.75	2.60	40.26	50.64	12.04	30.57	71.52	0.00	158.27	0.02	−9.87	−68.40	CaNa–HCO_3_SO_4_
				22.04.2010	19.5	361.94	2.61	37.63	45.01	12.88	30.45	65.72	0.00	166.74	0.00	−9.96	−70.34	CaNa–HCO_3_SO_4_
2	Wenchuan Well	103.77	31.63	10.06.2008	n.d.	n.d.	n.d.	n.d.	n.d.	n.d.	n.d.	n.d.	n.d.	n.d.	n.d.	n.d.	n.d.	n.d.
				21.10.2008	15.4	1569.81	4.21	71.65	182.01	128.73	47.39	878.08	0.00	248.12	−0.01	−8.37	−72.01	MgCa–SO_4_
				29.06.2009	15.5	1128.39	4.35	61.72	151.53	80.38	29.51	720.73	0.00	70.05	0.00	−10.14	−73.78	MgCa–SO_4_
				20.04.2010	10.8	1033.14	4.55	54.99	137.22	70.43	30.47	687.83	0.00	46.65	−0.01	−10.02	−73.11	MgCa–SO_4_
3	Lixian Well	103.59	31.48	09.06.2008	32.9	1579.54	5.64	35.95	252.78	103.76	8.15	862.32	0.00	310.70	0.00	−9.53	−80.88	CaMg–SO_4_
				22.10.2008	29.5	1195.08	5.00	37.29	182.66	78.59	7.82	812.50	0.00	68.47	−0.01	−11.75	−81.76	CaMg–SO_4_
				29.06.2009	27.1	1105.85	5.44	37.91	161.15	76.82	7.90	780.09	0.00	34.00	−0.01	−11.01	−82.13	CaMg–SO_4_
				20.04.2010	29.5	1025.28	5.72	36.15	146.64	69.97	7.48	741.96	0.00	14.63	−0.02	−11.62	−80.88	CaMg–SO_4_
4	Guergou Spring	103.17	31.46	11.06.2008	n.d.	349.33	2.33	91.11	5.24	0.80	2.58	21.76	0.00	223.52	0.00	−11.74	−116.81	Na–HCO_3_
				21.10.2008	58.0	246.71	2.23	60.76	4.74	0.83	2.45	21.33	0.00	150.62	−0.01	−14.42	−116.49	Na–HCO_3_
				28.06.2009	56.0	240.49	2.18	54.36	5.22	0.83	2.61	22.63	0.00	150.62	−0.03	−16.27	−116.99	Na–HCO_3_
				19.04.2010	55.8	151.69	2.19	29.82	4.76	0.82	2.31	22.16	0.00	88.13	−0.05	−16.34	−116.65	Na–HCO_3_
5	Jiuzhaigou Spring	103.00	31.52	11.06.2008	30.5	837.83	9.06	202.49	26.48	20.48	60.83	237.79	0.00	272.78	0.01	−14.01	−107.26	Na–HCO_3_SO_4_
				21.10.2008	32.1	813.62	9.15	189.00	26.01	20.66	64.16	225.51	0.00	272.34	0.01	−13.59	−106.10	Na–HCO_3_SO_4_
				28.06.2009	32.0	739.07	8.86	163.55	28.71	20.55	10.28	232.20	0.00	270.10	0.02	−14.57	−106.62	Na–HCO_3_SO_4_
				19.04.2010	27.3	728.04	8.75	150.98	26.73	20.41	9.65	241.18	0.00	267.84	0.00	−13.95	−106.54	Na–HCO_3_SO_4_

The MJF zone																		
6	Erdaohai Spring	103.32	32.73	08.06.2008	21.6	505.34	1.34	8.75	85.17	25.73	0.93	3.80	0.00	379.30	0.02	−13.72	−102.42	CaMg–HCO_3_
				22.10.2008	20.7	511.71	1.32	8.80	84.02	25.05	0.98	4.08	0.00	386.99	0.01	−14.39	−102.46	CaMg–HCO_3_
				29.06.2009	20.0	464.18	1.22	8.57	77.24	23.35	1.02	3.78	0.00	348.94	0.02	−14.57	−103.88	CaMg–HCO_3_
				21.04.2010	19.8	428.70	1.27	8.21	76.73	23.23	0.97	3.56	0.00	313.61	0.04	−14.39	−103.29	CaMg–HCO_3_
7	Kakagou Spring	103.68	32.98	08.06.2008	18.7	914.21	5.11	25.23	160.21	31.45	2.05	10.98	0.00	678.66	0.01	−13.56	−101.05	Ca–HCO_3_
				23.10.2008	19.7	876.14	4.92	24.08	154.12	30.74	1.93	10.86	0.00	648.69	0.01	−13.37	−101.92	Ca–HCO_3_
				30.06.2009	21.7	873.30	4.84	23.93	142.67	28.67	2.07	10.83	0.00	660.16	−0.01	−14.04	−100.46	Ca–HCO_3_
				21.04.2010	19.7	815.42	4.79	23.55	134.00	31.65	1.84	10.33	0.00	608.89	0.01	−14.12	−101.80	Ca–HCO_3_
8	Chuanpanqiao Spring	103.64	32.85	08.06.2008	8.8	1657.61	6.36	21.82	97.82	182.62	10.78	12.54	0.00	1324.27	−0.01	−14.20	−112.00	Mg–HCO_3_
				23.10.2008	9.6	1598.09	6.52	21.88	103.64	183.57	10.56	12.72	0.00	1258.08	0.00	−14.30	−112.48	Mg–HCO_3_
				30.06.2009	10.7	1557.18	5.89	21.68	103.79	175.29	10.55	11.54	0.00	1228.08	0.00	−15.34	−112.23	Mg–HCO_3_
				21.04.2010	10.7	n.d.	n.d.	n.d.	n.d.	n.d.	n.d.	n.d.	n.d.	n.d.	n.d.	−14.95	−113.33	n.d.
9	Songpan Well	103.60	32.65	08.06.2008	9.0	647.31	1.64	14.05	55.71	65.01	2.34	17.78	0.00	490.23	0.01	−13.25	−101.54	MgCa–HCO_3_
				23.10.2008	9.9	648.33	1.60	14.12	52.12	65.27	2.17	16.32	0.00	496.23	0.00	−13.43	−102.82	MgCa–HCO_3_
				30.06.2009	9.3	610.38	1.57	13.93	49.54	65.43	2.25	16.24	0.00	461.42	0.02	−13.20	−102.20	MgCa–HCO_3_
				21.04.2010	9.9	603.35	1.60	13.81	50.12	62.90	2.48	15.96	0.00	456.23	0.02	−13.77	−102.09	MgCa–HCO_3_

The XSHF zone																		
10	Ganzi Well	100.00	31.62	12.06.2008	27.8	441.46	3.42	49.07	38.84	19.07	4.04	44.27	0.00	281.36	0.00	−15.93	−125.50	NaCa–HCO_3_
				24.10.2008	29.8	447.22	3.53	47.97	37.51	19.97	3.94	43.44	0.00	289.54	−0.01	−16.48	−125.21	NaCa–HCO_3_
				27.06.2009	29.6	416.83	3.43	47.91	37.39	19.13	4.06	43.34	0.00	260.25	0.01	−16.17	−125.79	NaCa–HCO_3_
				18.04.2010	29.2	472.24	3.33	50.76	38.72	19.97	3.80	40.17	0.00	314.28	−0.01	−16.15	−125.75	NaCa–HCO_3_
11	Ganzi Spring	100.00	31.61	12.06.2008	43.1	735.04	8.95	154.67	23.95	16.42	6.38	62.15	0.00	459.17	0.01	−16.33	−124.72	Na–HCO_3_
				24.10.2008	41.5	731.75	9.08	150.20	23.13	16.34	6.19	60.61	0.00	461.22	0.00	−16.28	−124.30	Na–HCO_3_
				27.06.2009	43.0	695.04	9.17	146.66	22.34	16.44	6.00	61.79	0.00	427.90	0.01	−16.45	−126.00	Na–HCO_3_
				18.04.2010	41.4	706.99	9.29	138.30	21.87	16.42	6.17	63.67	0.00	447.27	−0.01	−16.29	−124.46	Na–HCO_3_
12	Guyi Spring	100.80	31.27	13.06.2008	35.5	2218.75	12.68	607.07	11.30	21.81	6.09	12.25	142.80	1399.60	0.01	−16.38	−130.98	Na–HCO_3_
				25.10.2008	31.3	2353.19	13.13	619.72	11.36	22.19	5.73	11.62	142.80	1524.39	0.00	−16.16	−131.17	Na–HCO_3_
				26.06.2009	33.2	2239.88	12.63	593.59	10.33	21.82	6.11	11.60	157.21	1424.29	−0.01	−16.97	−132.52	Na–HCO_3_
				18.04.2010	28.2	2366.42	12.63	619.91	10.01	21.31	5.95	12.35	108.56	1573.76	0.00	−16.56	−131.90	Na–HCO_3_
13	Longpugou Spring 1	101.24	30.98	13.06.2008	37.8	1180.32	7.16	310.06	11.00	8.23	13.83	6.83	0.00	820.21	0.01	−16.74	−137.69	Na–HCO_3_
				25.10.2008	37.2	1199.61	6.96	288.86	11.69	7.90	13.02	6.81	0.00	860.75	−0.01	−17.02	−136.73	Na–HCO_3_
				26.06.2009	39.2	1164.18	6.90	280.27	11.28	7.80	13.27	6.61	0.00	836.33	−0.01	−17.41	−137.71	Na–HCO_3_
				18.04.2010	36.5	1145.60	6.83	281.70	11.16	7.85	12.98	6.56	0.00	815.75	−0.01	−17.47	−138.90	Na–HCO_3_
14	Longpugou Spring 2	101.24	30.95	13.06.2008	44.5	1070.89	6.47	221.80	18.30	26.65	8.85	10.09	57.10	719.87	−0.02	−16.85	−134.42	Na–HCO_3_
				25.10.2008	42.5	927.46	6.93	205.03	18.90	26.11	8.28	9.78	0.00	650.41	0.02	−17.52	−134.80	Na–HCO_3_
				26.06.2009	42.5	900.81	6.67	198.93	17.43	25.73	8.39	9.33	0.00	634.33	0.02	−16.63	−133.90	Na–HCO_3_
				18.04.2010	43.2	919.25	6.48	176.77	18.84	25.93	8.53	9.63	0.00	671.47	−0.01	−17.61	−133.70	Na–HCO_3_
15	Bamei Spring	101.62	30.54	14.06.2008	55.2	1966.85	76.60	443.00	15.17	29.06	23.77	6.78	0.00	1368.29	0.01	−17.34	−135.61	Na–HCO_3_
				25.10.2008	55.6	1913.45	75.22	417.08	14.76	28.42	20.55	7.00	0.00	1346.62	0.00	−17.57	−134.51	Na–HCO_3_
				26.06.2009	56.0	2068.93	77.29	463.31	14.32	26.66	20.58	6.81	0.00	1456.10	0.00	−17.54	−135.81	Na–HCO_3_
				17.04.2010	55.6	2724.13	74.40	679.23	14.49	29.42	20.55	7.12	0.00	1894.64	0.02	−17.89	−134.41	Na–HCO_3_
16	Guanding Spring	101.96	29.98	15.06.2008	80.5	1306.05	41.23	396.79	13.16	13.21	336.16	51.96	11.34	440.26	0.02	−14.24	−117.01	Na–ClHCO_3_
				26.10.2008	80.0	1395.37	40.57	367.22	13.41	13.64	328.54	50.02	92.82	485.48	−0.03	−15.26	−118.02	Na–ClHCO_3_
				25.06.2009	83.0	1353.81	42.72	367.40	13.67	13.50	320.93	49.72	99.82	443.09	−0.02	−17.02	−127.74	Na–ClHCO_3_
				17.04.2010	83.0	1273.60	41.52	349.71	13.80	13.81	300.83	50.63	56.00	447.24	−0.01	−16.13	−123.72	Na–ClHCO_3_
17	Longtougou Spring	101.96	29.98	15.06.2008	70.8	2044.87	53.20	510.82	16.02	31.42	220.68	8.33	0.00	1200.36	0.01	−15.99	−128.69	Na–HCO_3_
				26.10.2008	70.2	2106.82	52.72	518.38	17.41	30.95	203.66	8.59	0.00	1270.92	0.00	−16.26	−127.78	Na–HCO_3_
				25.06.2009	73.1	2025.87	52.27	516.68	16.71	30.84	226.15	8.30	0.00	1171.25	0.01	−16.61	−128.57	Na–HCO_3_
				17.04.2010	70.2	2096.92	51.62	507.94	16.33	31.09	207.55	8.30	0.00	1270.92	0.00	−17.04	−130.94	Na–HCO_3_
18	Zheduotang Spring	101.86	30.01	15.06.2008	54.5	681.59	3.13	208.79	4.87	0.12	10.55	17.57	67.83	342.06	−0.01	−18.06	−138.80	Na–HCO_3_
				26.10.2008	53.8	705.52	3.14	219.83	4.38	0.12	10.57	17.31	85.68	338.08	−0.01	−18.21	−137.20	Na–HCO_3_
				25.06.2009	53.4	650.33	3.04	207.09	4.44	0.12	9.59	16.57	35.89	346.59	0.01	−18.64	−141.98	Na–HCO_3_
				17.04.2010	53.8	663.17	2.87	208.84	4.72	0.12	9.76	16.42	88.25	305.09	−0.02	−18.84	−139.88	Na–HCO_3_
19	Erdaoqiao Spring	101.95	30.09	15.06.2008	39.1	843.39	23.54	150.53	22.10	44.05	43.88	114.59	0.00	443.52	0.02	−14.50	−112.90	NaMg–HCO_3_
				26.10.2008	39.4	919.66	22.47	150.67	22.08	44.68	40.36	107.06	0.00	530.93	0.00	−14.54	−112.16	NaMg–HCO_3_
				26.06.2009	40.6	926.35	22.36	146.31	20.20	44.48	43.92	109.47	0.00	538.41	−0.02	−15.57	−115.14	NaMg–HCO_3_
				16.04.2010	40.6	935.65	23.67	157.07	20.93	43.76	40.97	111.59	0.00	535.44	0.00	−15.01	−115.35	NaMg–HCO_3_
20	Xinxing Spring	102.06	29.75	16.06.2008	45.6	1287.50	9.41	422.66	12.53	9.69	148.03	28.85	106.33	545.15	0.03	−15.90	−115.30	Na–HCO_3_
				27.10.2008	44.1	1167.77	9.68	365.04	12.75	9.43	144.03	24.65	0.00	597.19	0.05	−15.53	−115.83	Na–HCO_3_
				24.06.2009	42.8	1289.88	9.23	360.63	11.97	9.36	148.41	24.07	124.73	597.17	−0.02	−15.63	−114.96	Na–HCO_3_
				23.04.2010	48.2	1038.46	9.19	303.10	12.04	8.98	156.23	21.31	0.00	523.35	0.02	−15.21	−115.86	Na–HCO_3_
21	Gonghe Spring	102.11	30.62	16.06.2008	48.8	578.15	9.94	108.43	19.30	22.06	18.44	60.27	0.00	337.54	0.01	−11.91	−88.74	Na–HCO_3_
				27.10.2008	47.6	598.18	10.03	106.53	20.12	22.07	17.83	60.07	0.00	359.48	0.00	−12.40	−90.07	Na–HCO_3_
				24.06.2009	50.0	610.23	9.57	107.62	18.49	21.27	17.36	65.08	0.00	368.95	−0.01	−12.49	−89.29	Na–HCO_3_
				23.04.2010	44.8	585.91	9.41	111.76	19.32	21.47	17.85	64.11	0.00	339.32	0.01	−12.33	−89.00	Na–HCO_3_
22	Erhaoying Spring	102.03	29.59	17.06.2008	65.5	597.31	18.34	154.11	13.45	4.62	37.14	59.30	0.00	307.61	0.02	−14.22	−105.92	Na–HCO_3_
				27.10.2008	67.4	645.11	18.25	156.50	13.48	4.64	36.80	62.21	0.00	349.86	0.00	−14.31	−109.95	Na–HCO_3_
				25.06.2009	63.0	585.59	18.53	153.02	13.20	4.72	35.31	62.43	0.00	295.11	0.03	−15.13	−108.27	Na–HCO_3_
				24.04.2010	67.4	631.67	18.29	150.44	13.01	4.75	35.97	59.85	0.00	348.99	0.00	−15.27	−108.87	Na–HCO_3_
23	Tianwanhe Spring	102.14	29.49	17.06.2008	53.0	613.88	17.60	76.93	13.10	45.32	24.61	152.25	0.00	281.49	−0.01	−10.31	−79.22	NaMg–HCO_3_SO_4_
				25.10.2009	n.d.	632.23	16.92	73.37	12.74	45.71	23.42	139.62	0.00	317.73	−0.02	−10.04	−78.77	NaMg–HCO_3_SO_4_
				24.06.2009	54.0	611.73	17.58	79.12	12.81	43.62	24.91	145.24	0.00	286.45	−0.01	−10.50	−83.14	NaMg–HCO_3_SO_4_
				24.04.2010	53.2	644.79	17.44	79.11	12.71	44.44	25.65	145.44	0.00	317.73	−0.02	−10.59	−80.38	NaMg–HCO_3_SO_4_
24	Caoke Spring	102.10	29.39	18.06.2008	n.d.	n.d.	n.d.	n.d.	n.d.	n.d.	n.d.	n.d.	n.d.	n.d.	n.d.	n.d.	n.d.	n.d.
				28.10.2008	41.4	407.78	3.00	48.74	50.10	6.19	3.97	155.60	0.00	139.19	−0.02	−12.38	−88.30	CaNa–SO_4_HCO_3_
				24.06.2009	42.9	363.08	2.97	46.19	44.43	6.11	3.94	160.52	0.00	98.05	−0.01	−12.50	−91.09	CaNa–SO_4_HCO_3_
				24.04.2010	41.4	369.36	3.03	46.29	45.63	6.17	3.57	161.87	0.00	101.89	−0.02	−12.59	−90.94	CaNa–SO_4_HCO_3_
25	Shimian Well	102.22	29.44	18.06.2008	n.d.	n.d.	n.d.	n.d.	n.d.	n.d.	n.d.	n.d.	n.d.	n.d.	n.d.	n.d.	n.d.	n.d.
				28.10.2008	30.4	150.26	2.35	38.60	6.99	1.20	2.58	17.82	0.00	69.62	0.00	−11.08	−87.15	Na–HCO_3_
				23.06.2009	31.3	149.10	2.39	37.61	6.46	1.23	2.44	17.29	0.00	70.79	0.00	−11.28	−90.21	Na–HCO_3_
				24.04.2010	31.2	137.77	2.34	35.14	6.86	1.20	2.35	17.16	0.00	62.48	0.01	−10.51	−88.94	Na–HCO_3_

The ANHF zone																		
26	Gongyihai Spring	102.39	29.02	18.06.2008	56.9	n.d.	n.d.	n.d.	n.d.	n.d.	n.d.	n.d.	n.d.	n.d.	n.d.	−14.23	−108.54	n.d.
				28.10.2008	54.1	320.27	3.34	109.14	2.37	1.00	1.91	46.17	0.00	82.11	0.00	−14.19	−108.67	Na–HCO_3_SO_4_
				23.06.2009	55.4	306.82	3.37	100.47	2.40	1.01	1.99	48.14	0.00	81.95	−0.01	−14.83	−110.47	Na–HCO_3_SO_4_
				24.04.2010	56.4	323.92	3.23	113.91	2.44	1.02	3.80	47.98	0.00	88.81	0.02	−14.82	−110.20	Na–HCO_3_SO_4_
27	Chuanxin Spring	102.36	28.26	19.06.2008	n.d.	462.71	4.80	86.08	15.24	11.55	5.52	12.67	0.00	325.96	−0.01	−13.46	−106.54	Na–HCO_3_
				28.10.2008	n.d.	449.48	4.25	86.72	15.29	11.45	5.18	12.44	0.00	311.73	0.45	−13.32	−106.98	Na–HCO_3_
				23.06.2009	49.0	483.93	4.23	85.56	13.75	10.18	4.66	12.00	0.00	351.73	−0.04	−14.51	−108.29	Na–HCO_3_
				24.04.2010	45.1	456.34	4.23	85.46	13.21	9.52	4.72	12.74	0.00	325.14	−0.02	−13.41	−107.37	Na–HCO_3_
28	Taihe Spring	102.14	27.91	19.06.2008	21.1	226.41	0.69	58.65	4.03	0.76	1.99	12.76	0.00	146.70	0.01	−13.37	−106.65	Na–HCO_3_
				29.10.2008	21.1	206.70	0.66	53.56	4.02	0.00	1.91	12.72	0.00	132.20	0.00	−13.31	−106.32	Na–HCO_3_
				n.d.	n.d.	n.d.	n.d.	n.d.	n.d.	n.d.	n.d.	n.d.	n.d.	n.d.	n.d.	n.d.	n.d.	n.d.
				n.d.	n.d.	n.d.	n.d.	n.d.	n.d.	n.d.	n.d.	n.d.	n.d.	n.d.	n.d.	n.d.	n.d.	n.d.
29	Xichang Well	102.15	27.91	19.06.2008	22.3	261.87	0.31	78.93	2.08	0.81	21.78	15.22	0.00	137.09	0.01	−13.78	−106.89	Na–HCO_3_
				29.10.2008	n.d.	229.28	0.32	71.76	2.15	0.85	21.32	15.22	0.00	112.05	0.02	−13.23	−106.64	Na–HCO_3_
				22.06.2009	24.2	238.13	0.33	69.68	2.00	0.81	21.38	15.65	0.00	123.05	0.00	−13.24	−106.07	Na–HCO_3_
				n.d.	n.d.	n.d.	n.d.	n.d.	n.d.	n.d.	n.d.	n.d.	n.d.	n.d.	n.d.	n.d.	n.d.	n.d.
30	Zhuhexiang Spring	102.93	28.07	19.06.2008	49.8	256.45	4.65	54.62	13.18	2.42	3.99	11.97	0.00	161.91	0.02	−12.62	−100.01	Na–HCO_3_
				29.10.2008	49.7	248.22	4.57	51.93	13.39	2.35	3.91	12.22	0.00	154.30	0.00	−12.67	−100.50	Na–HCO_3_
				22.06.2009	49.8	244.72	4.27	50.23	13.75	2.36	3.96	12.05	0.00	152.89	0.00	−13.53	−99.12	Na–HCO_3_
				26.04.2010	48.0	255.93	4.27	50.17	13.26	2.44	3.80	12.31	0.00	167.97	0.00	−12.86	−100.54	Na–HCO_3_
31	Lizhouzhen Spring	102.24	28.09	19.06.2008	n.d.	331.37	14.12	54.16	30.00	0.00	5.52	11.33	0.00	212.12	0.01	−14.48	−110.35	NaCa–HCO_3_
				29.10.2008	48.1	326.67	14.66	52.79	30.77	0.00	5.38	11.34	0.00	210.92	0.02	−14.71	−110.23	NaCa–HCO_3_
				22.06.2009	49.0	323.38	13.37	48.45	29.36	0.00	6.14	10.93	0.00	210.00	−0.01	−14.11	−110.74	NaCa–HCO_3_
				25.04.2010	48.6	321.73	14.08	49.49	29.49	0.00	5.45	11.06	0.00	209.92	0.00	−14.63	−110.06	NaCa–HCO_3_
32	Luojishan Spring	102.54	27.40	20.06.2008	n.d.	1212.86	49.31	208.79	81.48	29.43	136.74	257.26	0.00	449.06	0.00	−11.76	−96.43	Na–HCO_3_SO_4_
				29.10.2008	43.7	1172.03	46.80	213.50	81.42	28.43	135.47	255.58	0.00	407.06	0.01	−11.64	−96.10	Na–HCO_3_SO_4_
				22.06.2009	43.8	1150.63	46.67	213.09	71.12	26.07	135.76	244.07	0.00	412.82	0.01	−13.41	−96.20	Na–HCO_3_SO_4_
				25.04.2010	43.8	1127.84	43.86	200.41	73.26	26.02	135.37	252.58	0.00	395.58	0.00	−13.18	−96.77	Na–HCO_3_SO_4_

The “n.d.” represents samples not analysed.
